# Leading Age-Friendly University Initiatives at a Research Intensive University in Manitoba, Canada

**DOI:** 10.1093/geroni/igaa057.1791

**Published:** 2020-12-16

**Authors:** Michelle Porter

**Affiliations:** University of Manitoba, Winnipeg, Manitoba, Canada

## Abstract

When Dublin City University introduced the Age-Friendly University (AFU) principles in 2012, the Centre on Aging at the University of Manitoba had been fully immersed in age-friendly cities research, so it was a natural for it to champion the University becoming more age-friendly. As a university-wide research centre, at a research intensive university, the Centre on Aging has built on its strengths to advance the AFU movement. Related to its goals on research, knowledge mobilization, training, and partnerships, it has been able to work with its many Research Affiliates, students and community/university partners to tackle new AFU initiatives. Recent activities have included: an awareness raising workshop/showcase, discussions with Research Affiliates about potential new degree course offerings, an environmental scan of post-secondary inter-generational possibilities, a home-sharing project, workshop development on older learners in the classroom, and provincial community workshops and consultations on communicating about aging and healthy aging. Part of a symposium sponsored by Directors of Aging Centers Interest Group.

